# Dog–Human Play, but Not Resting Post-Learning Improve Re-Training Performance up to One Year after Initial Task Acquisition in Labrador Retriever Dogs: A Follow-On Study

**DOI:** 10.3390/ani10071235

**Published:** 2020-07-21

**Authors:** Nadja Affenzeller

**Affiliations:** Department of Companion Animals, Clinical Unit of Internal Medicine Small Animals, University of Veterinary Medicine Vienna, Veterinärplatz 1, 1210 Vienna, Austria; nadja.affenzeller@vetmeduni.ac.at

**Keywords:** dog, memory, consolidation, play, activity, rest

## Abstract

**Simple Summary:**

Situations that are emotional and arousing can have an effect on the memorability of events. The beneficial effect of dog–human play immediately after learning a new task has recently been shown to improve training performance in companion dogs (Labrador Retrievers) when required to solve the same task 24 h later. This follow-on study re-trained the same dogs after a period of one year in the same two-choice discrimination paradigm. Analyzed factors included: the age of the dogs, the effect of the trainer identity, training performance in the previous study, heart rate, and the number of trials and errors to meet the re-training criterion. The results show that all dogs relearned the task; however, dogs from the dog–human play group needed significantly fewer trials and made significantly less errors when compared to the control group. To the author’s knowledge, this is the first evidence that post-training activity may influence memory in dogs up to 1 year after the initial task acquisition. However, when interpreting the overall results, the limitations due to the low sample size must be taken into account.

**Abstract:**

Arousing and emotional situations can improve cognitive performance and the memorability of events. Recently, the enhancement of training performance in Labrador Retriever dogs through 30 min of dog–human play immediately after acquiring a novel task, when compared to a resting period, was demonstrated. This follow-on study used the same pseudo-randomized, counterbalanced, between-subject study design, and 11 Labrador Retrievers were re-trained in the identical two-choice discrimination paradigm after a period of 1 year. The playful activities group needed significantly less trials and made significantly less errors to successfully reach the re-training criterion (Mann–Whitney U test, critical value of U at *p* < 0.05 is 5, U = 5, Z = 1.73, *p* = 0.04 and U = 4.5, Z = 1.8, *p* = 0.03, respectively). Following model simplification of a multiple factor/covariate general linear model analysis, the type of intervention, the number of trials needed to re-learn the task after 24 h, the average heart rate during the intervention a year ago, and age were significantly correlated to the number of trials and errors needed to resolve the task. A significant difference due to intervention allocation (heart rate during the intervention, trials needed to re-learn the task after 24 h) between the groups was confirmed. Age did not significantly differ between the groups; nevertheless, the effects of ageing cannot be fully excluded, given the low sample size. No effects of the trainer and of the cortisol concentrations (of the previous year) were observed. This is the first evidence that post-training activity may influence memory up to 1 year after task acquisition.

## 1. Introduction

Scientific interest in the field of learning and memory has grown recently [1,2]. However, most studies are performed on humans, non-human primates, and rodents. Information about companion animals, especially dogs, is still very scarce. This is surprising given that dogs are trained to fulfil specific tasks; for instance, detection of explosives and drugs in the professional sector or for guide- and assistance dogs in the private sector. Given the time and money invested in such training [3], further information about factors influencing memory and, ultimately, training performance would be tremendously valuable.

So far, the two most promising intervention factors are the active role of sleep and positive emotional situations that lead to arousal. Both interventions have recently been shown to improve cognitive performance and the memorability of events in dogs, in the sense of improved training performance when induced immediately post-learning of a newly acquired task [4,5].

The underlying mechanisms of sleep to enhance memorability are not fully understood yet. However, it is believed that learning affects the EEG spectrum, and that certain spectral features, amongst them spindle density, are related to post-sleep performance improvement [2].

In humans, it is well known that emotionally arousing, stressful situations can create long-lasting memories. When considering an evolutionary point of view, it is hypothesized that this might serve an adaptive function. Being able to remember important features of a specific context can help to be better prepared in a similar future context [6]. The role and interplay of different neuroanatomical structures, neural pathways, and activating and deactivating neurotransmitters and neuromodulators have been extensively explored to explain underlying mechanisms (please see review [7] for further details). Briefly, it is thought that beta-adrenergic activation and the release of specific stress hormones enhance memory consolidation and lead to an increase in memorability through the facilitation of memory recall [7]. More specifically, it has been shown that adrenaline, noradrenergic activation of the amygdala, and beta-adrenergic receptor activation are essential to improve the memorability of stressful events [8,9]. Most importantly, this activation of the sympathetic nervous system can be induced by both aversive stimuli [7] and pleasant stimuli [10]. In summary, the combination of beta-adrenergic activation and the release of adrenal hormones are thought to be responsible for enhancing the memorability of emotionally arousing contexts [7].

Recently, it was demonstrated that training performance was enhanced in Labrador Retriever dogs that have been engaged in 30 min of dog–human play immediately after successfully acquiring a two-choice object discrimination task post-training [4]. This study investigated the effects of an acute, post-learning, positively arousing event in the form of playful activities, which included exploring the environment while being walked to an off lead area, where all dogs were engaged in games, such as fetching a ball, running after Frisbees, and playing tug-of-war, depending on each dog’s preferred play style. A pseudo-randomized, counterbalanced, between-subject study design was used, and 16 Labrador Retriever dogs ranging from 1 to 9 years of age were able to successfully finish the training. A range of factors including age, sex, training experience, and trials to meet the training criterion were analyzed. All dogs in the control group were asked to rest by settling on a dog bed, while the researcher engaged the owner in a conversation to prevent further attention or interference with the dog. The results demonstrated that playful activities with a human researcher post-learning improved training performance, evidenced by the fewer trials needed to relearn the task 24 h later (dog–human play group: mean number of trials 26 ± 6; resting group: mean number of trials 43 ± 19, effect size d = 1.2). Indeed, it has recently been shown that interaction in the form of play can induce a positive affective state in both dogs and humans, with the additional effect of play being considered intrinsically rewarding [11].

Assessing the longevity of a specific intervention is an important aspect when studying the memorability of events. However, to the author’s knowledge, no data on this aspect, beyond re-testing a couple of weeks after the initial task acquisition, have been published in dogs, so far [5,12]. Techniques that could enhance memory and improve training efficacy long-term would be tremendously valuable, especially in dogs who are extensively trained to aid humans. 

Hence, the aim of this follow-on study was to determine if a single intervention of an induced positive affective state in dogs (in the form of dog–human play) has a long-lasting effect on memorizing a previously learned task. To do this, the same study population of dogs were re-recruited to solve the same two-choice object discrimination paradigm after a period of approximately 1 year.

## 2. Materials and Methods 

### 2.1. Study Design

This study was a follow-on study, which is based on the original between-subject design, following a methodologically standardized approach with a pseudo-randomized object location and two groups of dogs, balanced for trained object, intervention type, age, and cognitive testing experience [4].

The project has been discussed and approved by the institutional ethics and welfare committee, in accordance with GSP guidelines and the national legislation of the University of Veterinary Medicine Vienna, Austria under ETK- 08/09/2015, and also met the ethical guidelines of the University of Lincoln, United Kingdom.

### 2.2. Study Group

Eleven purebred Labrador Retrievers (out of an initial 16) were recruited to participate in this follow-on study (see Table 1 for individual information). The other 5 dogs were lost for follow-up. Three males (1 intact, 2 neutered) and 8 females (3 intact, 5 spayed), ranging from 3 to 10 years, were tested at the Riseholme Campus, University of Lincoln, UK. All dogs were privately owned pet dogs, still staying with the same owners, and informed consent was obtained once more. All dogs were reported as being healthy by their owners, without the need for medication. None of the dogs had been trained on the same or a different two-choice object discrimination task since the original study.

### 2.3. Summarised Procedure

In the previous study (with 16 dogs), after initial acquisition of the two-choice discrimination task (OD training) on day 1, either a playful activity intervention or a resting period took place (see [4] for details). Briefly, after meeting the criterion in the OD training, either a 30-min resting period or a playful activity consisting of a 10 min walk, 10 min off lead dog–human play, and another 10 min walk followed. The dogs in the resting group were asked to settle on a dog bed, while the researcher engaged the owner in a conversation to prevent further attention or interference with the dog. However, when lying their head on the floor, dogs were called by their name and/or touched to prevent them from falling asleep. The dogs in the playful activity group were allowed to explore the surroundings, while being walked to a fenced in area (20.5 m × 33.5 m). Dog–human play consisted of fetching a ball, running after Frisbees, and playing tug-of-war, depending on each dog’s preferred play style, reported by its owner or chosen by the individual dog.

**Figure 1:**
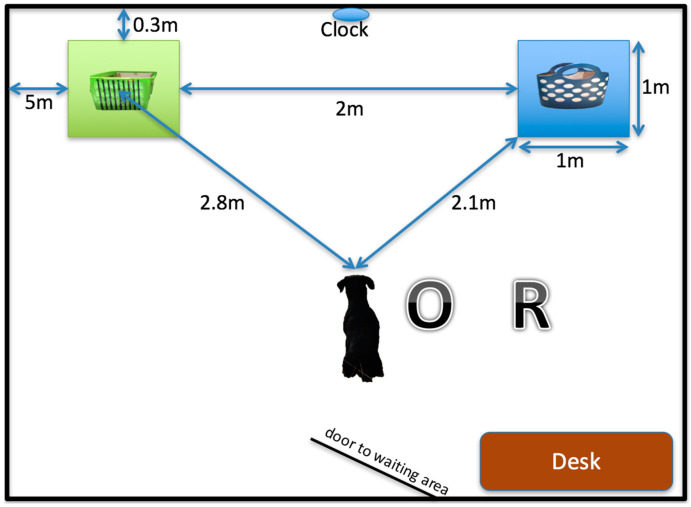
Setup and dimensions of the testing area including the walking distance. O: designated area of the owner, R: designated area of the researcher.

In this follow-on study, dogs were re-trained based on their previous object and group allocation after 1 year of the initial task acquisition. Each dog was trained to correctly indicate one out of two objects (blue, dotted, woodchip filled basket or green, striped, cat litter sand filled box), undergoing the same training procedure until the training criterion was met again; each session consisted of 10 trials and the success rate was determined to be ≥80% in two consecutive sessions. However, to avoid a location and experimenter bias, all dogs were retested in a different room, (see Figure 1 for details and dimensions) and trained by two different researchers, unknown to the dogs and their owners. 

During the training, all dogs wore a Polar^©^ RS800CX heart rate monitor (Polar Electro Oy, Kempele, Finland) consisting of a watch receiving and storing the data, and an electrode belt and transmitter (Polar, Wear Link Smart Fabric sensor W.I.N.D^©^, Kempele, Finland), which has been shown to reliably measure heart rate in dogs [13]. This device measures heart beats at a frequency of 1000 Hz with a transfer rate of 2.4 GHz between the belt and the heart rate monitor. Ultrasound gel (Konix^©^ Ultrasound Gel, Turkuaz Ltd., Istanbul, Turkey) was used to promote conductivity. The heart rate data were transmitted at the end of the training to a laptop computer using the Polar software Protrainer 5^©^. Each dog’s heart rate data were exported as a text file into Kubios^©^ HRV software Version 2.2. (University of Eastern Finland, Kuopio, Finland). 

### 2.4. Training Details

All dogs were trained under daylight conditions in a room on solid anti-slip flooring with water freely available at all times. All dogs were allowed to freely explore the room for at least 5 min, during which time the training objects were not present. Then, the heart rate monitor electrode belt was strapped around the chest of the dog and fixed with another strap (3M^©^ Vetrap Bandaging Tape, 3M Animal Care Products, St. Paul, MN, USA) around the shoulders. The transmitter was placed ventrally, with the electrodes positioned on each side of the sternum. Ultrasound gel was applied liberally between electrodes and fur until a signal was transmitted to the watch. 

All dogs had to pass a pre-training criterion before starting with the OD training. All dogs were re-trained to go to an object (a Jar: 15 × 11 × 17 cm) to re-establish a release cue, “Go”. The jar was placed in an alternating order on either of the same two spots where the OD training objects were later placed. On the first two occasions, a piece of sausage was visibly placed by the researcher on top of the jar, to motivate the dogs to approach it. The criterion was met when the dogs had at least two paws within 0.5 m of the jar in 4 out of 5 trials. When the dogs had at least two paws within 0.5 m of the jar, they were rewarded with a clicking noise that was followed by a piece of sausage. A dog needing more than 10 sessions with 5 trials each to meet the criterion was excluded from participating further.

After passing the pre-training criterion, the dogs were trained in the same 2-choice discrimination paradigm to differentiate between the same two objects, differing in multiple dimensions: odor (cat litter vs. woodchip), pattern (black stripes vs. white dots), size and shape (box vs. basket), and color (light green vs. dark blue), as shown in Figure 1. The blue basket with white dots was filled with woodchips (Durstons^©^ Large Chip Bark, Durston Garden Products Limited, Somerset, UK), and the green box with black stripes was filled with cat litter (Msavers^©^ Cat Litter, Morrison Supermarkets PLC, Bradford, UK), each to a depth of approximately 5 cm. Each object was placed in the middle of a 1 × 1 m cardboard square covered in a cotton towel, the color of which corresponded to the object color. Each dog was trained to correctly indicate one out of the two objects, the object assigned to each dog was the same as in the previous year. 

To do this, the owner was asked to walk the dog from the waiting area to the designated starting point, and then stand next to the dog, looking ahead at a clock placed centrally on the wall (see Figure 1). The owner and the researcher wore dark sunglasses at all times. The researcher stood in a marked, designated area on the right hand side of the owner, and cued the owner to release the dog on the “Go” command after the dog continuously looked towards the setting for at least 2 s. On the first two occasions, a piece of sausage was visibly placed inside the correct object, which was placed on the right and left designated area (both sides baited for every dog), to motivate the dogs to approach it after the release cue. After the dog approached an object, the researcher used either a click sound followed by a reward (one piece of pork or chicken sausage fed by hand from the researcher) or a spoken “Wrong” in a neutral voice. After each trial, the owner and the dog returned to the waiting area until being called back in by the researcher for the next trial. The location of the objects was pseudo-randomized using the free online software Research Randomizer [14], such that each object was presented on the left and right side an equal number of times, but not on the same side for more than two consecutive trials, to prevent the development of a side bias by the dogs. The dog was considered to have made a choice when 2 paws were placed on the cardboard square of the object. No choice was defined as the dog not having two paws on either square within 30 s after the release cue. Trials where no choice was made were not counted as correct or incorrect, instead the same trial was repeated. Three consecutive no choices were followed by a break. Another 3 consecutive no choices after a break led to the exclusion of the dog from the study. After a correct choice, the researcher continued with the next trial; whereas after a wrong choice, the same trial was presented again until the dog made a correct choice. Three consecutive wrong choices in the same trial resulted in the owner walking the dog over to the correct object, which was followed by the next trial. The criterion was met when the dog had an 80% or higher correct choice in two consecutive sessions, each session consisting of 10 trials. After each session, breaks of 5 to 10 min were given, during which the dogs were allowed to relax in the surrounding area.

### 2.5. Data Analysis

The total number of trials and errors needed to meet the training criteria, and the number of no-choices were counted. The relative training performances comparing re-learning after 24 h and re-learning after 1 year for each individual dog were calculated using Microsoft Excel^©^ (Microsoft Office 2016, Redmond, WA, USA). 

Given the low number of dogs in each group in this follow-on study, the non-parametric Mann–Whitney U test was used. One-tailed results and the critical value of U at *p* < 0.05 were presented. In addition, Z values and the correspondent *p* values were additionally presented for readers, not used to interpret results based on U values only. The median values and the interquartile ranges were reported.

Average heart rate during re-training was calculated using Kubios^©^ HRV software Version 2.2. (University of Eastern Finland, Kuopio, Finland). Average heart rate data results were reported as mean ± SD. 

Statistical analysis was performed using Minitab^©^ Statistical Software (updated version 19.2020.1, State College, PA, USA). A multiple factor/covariate General Linear Model analysis, including analysis for multicollinearity of independent variables, was performed, followed by backwards stepwise simplification, where non-significant highest to lowest order main effects were excluded first (alpha to remove value: 0.1). The residuals had to be normally distributed. The factors and the covariates included age, the type of intervention, the researcher, the training performance in the previous study (the numbers of trials needed to re-learn the task after 24 h), the average heart rate (during the intervention), and the cortisol concentration levels (taken after the intervention, for details see [4]). The number of trials and errors to meet the re-training criterion were dependent variables.

The dogs that failed to meet the criterion for pre-training or OD training were excluded from further statistical analysis. A binomial test was conducted to identify the criterion for an individual’s performance that was significantly above chance level (16 out of 20, 80% or higher, *p* = 0.01) [15].

## 3. Results

### 3.1. Results on Training Procedure

No dog was excluded from the study population; all dogs passed the pre-training criterion within one session. In addition, all dogs reached the training criterion successfully. No “no choices” were recorded.

### 3.2. Results on Absolute Number of Trials and Preexisting Group Differences

There were no overall effects of the type of intervention, the researcher, the training performance in the previous study (the numbers of trials needed to re-learn the task after 24 h), the average heart rate (during the intervention), the cortisol concentration levels (taken after the intervention in the previous year), and age on the absolute number of trials to reach the training criterion (General Linear Model: *p* > 0.1; adjusted R^2^: 49.6%). However, following model simplification, the type of intervention (F_1,10_ = 7.68, *p* = 0.03), the number of trials needed to re-learn the task after 24 h (F_1,10_ = 6.25, *p* = 0.047), the average heart rate during the intervention a year ago (F_1,10_ = 8.11, *p* = 0.03), and age (F_1,10_ = 10.98, *p* = 0.02) were significantly correlated to the absolute trial number. The adjusted R^2^ improved to 55.1%.

**Figure 2:**
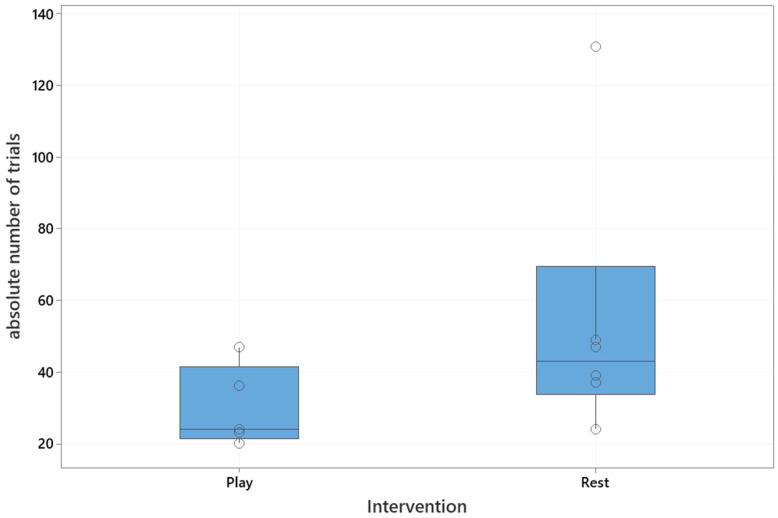
Boxplot of the absolute number of trials needed to reach the training criterion based on intervention. Boxplots show the median and interquartile range from the 25th to 75th interquartile. Open circles represent individual dogs. Play: playful activities group, Rest: resting group.

The median absolute number of trials to reach the training criterion was 37 (interquartile range: 24–47). The median number of trials in the playful activities group was 24 (interquartile range: 22–42), and in the resting group 43 (interquartile range: 34–70), which is shown in Figure 2. The playful activities group needed significantly less trials to reach the training criterion (Mann–Whitney U test, U = 5, critical value of U is 5, Z = 1.73, *p* = 0.04). 

A significant difference between the remaining individuals in each group after the intervention a year ago, with respect to the number of trials needed to relearn the task after 24 h, was confirmed (Mann–Whitney U test: U = 2.5, critical value of U is 5, Z = 2.19, *p* = 0.01). The median number of trials to re-learn the task after 24 h in the playful activities group was significantly lower when compared to the resting group (playful activities group: 23, interquartile range: 22–30; resting group 50, interquartile range: 41–63). 

The same is true for the average heart rate during the intervention between the remaining individuals of each group; a significant difference between the two groups was found. The playful activities group had significantly higher average heart rates during the intervention (Mann–Whitney U test: U = 0, critical value of U is 5, Z = −2.65, *p* = 0.004; mean average heart rate of the playful activities group: 143 ± 20 and the resting group 82 ± 21, respectively).

### 3.3. Results on Total Number of Errors

There were no overall effects of the type of intervention, the researcher, the training performance in the previous study (the numbers of trials needed to re-learn the task after 24 h), the average heart rate (during the intervention), the cortisol concentration levels (taken after the intervention in the previous year), and age on the total errors to reach the training criterion (General Linear Model: *p* > 0.1; adjusted R^2^: 55.6%). However, following model simplification, the type of intervention (F_1,10_ = 9.29, *p* = 0.02), the number of trials needed to re-learn the task after 24 h (F_1,10_ = 6.83, *p* = 0.04), the average heart rate during the intervention a year ago (F_1,10_ = 11.27, *p* = 0.01), and age (F_1,10_ = 12.56, *p* = 0.01) were significantly correlated to the total number of errors. The adjusted R^2^ improved to 62.4%.

**Figure 3:**
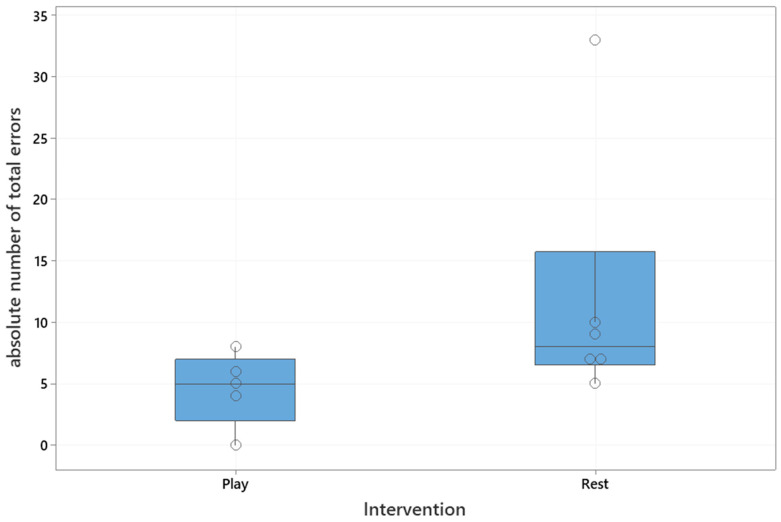
Boxplot of the absolute number of total errors to reach the training criterion based on intervention. Boxplots show the median and interquartile range from the 25th to 75th interquartile. Open circles represent individual dogs. Play: Playful activity group, Rest: resting group.

The playful activities group made significantly less total errors than the resting group (Mann–Whitney U test, U = 4.5, critical value of U is 5, Z = 1.8, *p* = 0.03), which is shown in Figure 3. The median number of total errors for the playful activities group was five (interquartile range: 2–7), and for the resting group was eight (interquartile range: 7–16).

### 3.4. Additional Results

No significant effect was found of the researcher and the cortisol concentration levels post-intervention in the previous year on the absolute numbers of trials and the absolute numbers of errors to re-learn the task 1 year later (*p* > 0.1).

In addition, there was no significant difference between groups in the length of time passed before re-testing (re-testing interval: playful activities group: median 12 months; interquartile range: 12–18; resting group: 15.5 months, interquartile range: 12–17, Mann–Whitney U test U = 10, critical value of U is 5, Z = 0.82, *p* > 0.1).

The mean average heart rate during re-training did not significantly differ between the groups (playful activities group: median 97 bpm, interquartile range: 89–114; resting group: median 112 bpm, interquartile range: 96–120; Mann–Whitney U test U = 10, critical value of U = 5, Z = 0.82, *p* > 0.1).

There was no significant difference in age between the playful activities group and the resting group (Mann–Whitney U test, U = 5.5, critical value of U is 5, Z = 1.64, *p* = 0.051). The median age of the resting group was 7.5 years (interquartile range: 5 to 9), and 4 years (interquartile range: 3–6) for the playful activities group.

### 3.5. Results on Individual Training Curves and Training Performances

**Figure 4:**
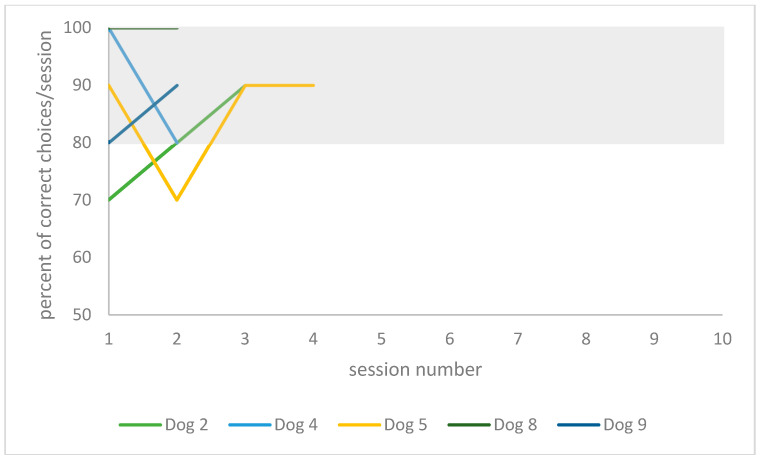
Individual training curves of dogs assigned to the playful activities group. The grey rectangle highlights the successful training criteria area (≥80% in two consecutive sessions).

**Figure 5:**
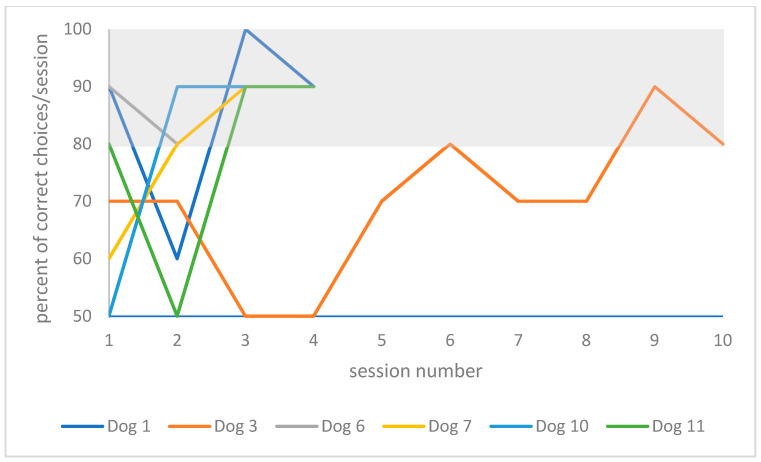
Individual training curves of dogs assigned to the resting group. The grey rectangle highlights the successful training criteria area (≥80% in two consecutive sessions).

The individual training curve of each dog is presented in Figure 4 for the playful activities group, and in Figure 5 for the resting group.

Individual training performance data over the one-year period are presented in Table 2. When comparing training performances between re-learning 1 year later to re-learning on day 2 in the playful activities group, one dog (dog 8, 3 years) was able to improve his performance by 9% (20 instead of 22 absolute trials), and one dog’s performance did not change (dog 9, 4 years old). The youngest and oldest dogs in this group deteriorated the most in their performances, (57% of trial number increase for dog 2, 3 years old, and 31% for dog 5, 7 years old). In the resting group, two dogs were able to improve in their performance (by 37%, 37 instead of 59 trials for dog 7, 6 years old; and by 67%, 24 instead of 73 trials for dog 6, 3 years old). One dog’s performance did not change (dog 1, 8 years old), and two dogs deteriorated in their performance (152% of trial number increase for dog 3, 8 years old; and 70% for dog 10, 10 years old).

When comparing training performances between the initial learning on day 1 to the day after (24 h later), all five dogs in the playful activities group improved (between 40% and 70%, respectively). In the resting group, four dogs improved (between 26% and 58%) and two dogs deteriorated in their performance (by between 6% and 18%). However, there was no statistically significant difference between the individuals of each group when comparing the absolute number of trials needed to learn the task on day 1 (Mann–Whitney U test: U = 10, critical value of U is 5, Z = 0.82, *p* > 0.1).

When comparing training performance between the initial learning on day 1 to 1 year later, all dogs of the playful activities group improved (a relative decrease in trial numbers of 46–65%). In the resting group, four dogs (dog 6, 3 years old; dog 7, 6 years old; dog 10, 10 years old; and dog 11, 7 years old) improved (a relative decrease in trial number of 47–73%). Two dogs deteriorated in their performance (18% relative trial number increase, dog 1, 8 years old; 87% relative trial number increase, dog 3, 8 years old).

## 4. Discussion

The current study was designed to explore the longevity of the previously reported positive effect of playful activities, in the form of dog–human play, after learning a new task in pet dogs. To the author’s knowledge, this is the first evidence that post-training activity may influence memory in dogs up to 1 year after the initial task acquisition. Dogs of the playful activities group needed a significantly lower number of trials to solve the identical object discrimination task learned the year before and made significantly less errors to reach the training criterion. This suggests that playful activities with a human for 30 min post-learning affected long-term memory, not only after 24 h [4], but, potentially, also up to 1 year later. 

However, it needs to be highlighted that the number of recruitable participants was limited to 11 dogs (five in the playful activities group and six in the resting group). This small sample size is one of the main limitations of this follow-on study, and results need to be interpreted considering this circumstance. Hence, this follow-on study should be seen in the light of an exploratory data character.

One main assumption of this follow-on study was that the remaining dogs in each group differed because of the previous training intervention experience that they were randomly allocated to, rather than because of individual learning capacities, changes thereof within the last year, or other factors, such as the experimenter or the time passed between re-testing. Indeed, the trials needed to relearn the task on day 2 and the average heart rate during the intervention on day 2 significantly affected training performance 1 year later. This finding is not surprising when considering that all eight dogs of the original study [4], but also the remaining five dogs of this follow-on study in the playful activities group, needed significantly fewer trials to solve the discrimination task on day 2 (24 h after the intervention had taken place on day 1). In addition, it was confirmed that all dogs of the playful activities group had significantly higher average heart rate levels due to their dog–human play intervention on day 1. Hence, it is concluded that these findings support the assumption that the remaining dogs in each group differed based on their previous training performance on day 2, which was caused by the dog–human play intervention itself. All of these factors were still affecting training performance 1 year later.

The intervention type itself and, also, age, significantly affected both the absolute trial number and the absolute error number 1 year later. Despite there not being a statistically significant difference between the two groups, this difference could also be classified as marginally non-significant (Mann–Whitney U test, U = 5.5, critical value of U is 5, Z = 1.64, *p* = 0.051). The resting group was older, with a median age of 7.5 years (interquartile range: 5 to 9), when compared to 4 years (interquartile range: 3–6) for the playful activities group. 

The current object discrimination literature shows inconsistent results when analyzing age effects on discrimination learning. Dog populations, age ranges, training task details, and learning criterion vary largely within the literature; this makes direct comparisons difficult [16]. One of the most recent studies in this field was able to show that younger dogs (2–6 years old) were faster in the initial task acquisition when compared to older dogs (>8 years old) [17]. However, in the same study, no age effect was seen between the groups after a retention phase of 5 min had passed, and dogs were retested in 10 trials identical to the training trials. This confirms findings of another study, which compared younger dogs to dogs older than 8 years, where an age effect was not detected after a retention phase of 2 weeks, following the initial discrimination learning [12]. The oldest dog in this cohort was 10 years old, and deficits in learning a discrimination task have been reported in dogs aged >10 years [18]. However, it was also reported that 25% of dogs in this age cohort showed no deficit in their training performance. When assessing the training performance of this follow-on study population, the oldest dog (10 years old) met the criterion with 57% less trials after 1 year, when compared to the initial task acquisition on day 1. When looking at the training performances of all dogs, only two dogs (both in the resting group) required more trials when compared to their initial training success on day 1. However, 47 (a relative deterioration of 18%) and 131 (a relative deterioration of 87%) trials to successfully reach the training criterion were still within the training performance range in the previous year. The mean absolute trials to reach the training criterion on day 1 of all previous 16 dogs was 83 (SD 39), with the minimum of 37 and the maximum of 181; the dog who took the most trials to initially learn the task was 2 years old (see [4] for details of the full group). When interpreting overall training performances, considering the improvement of all other dogs (including two 7 year old dogs and the 10 year old dog), an obvious effect of age was not observed; however, given the limitation of the low sample size, its potential for playing a role cannot be fully excluded. It also needs to be pointed out that all dogs successfully re-learned the task, and no dog had to be excluded from the study population. Hence, it is concluded that the overall good performance indicates that the training criterion was set sufficiently high, meaning dogs were indeed able to remember the discrimination training from the previous year.

Most interestingly, it has been shown that performance in relation to simplicity/difficulty of a discrimination task is influenced by age. In simple visual discrimination tasks, aged dogs performed just as well as young dogs [19]. More difficult tasks, such as discrimination learning that is followed by reversal learning, have been shown to be a more sensitive measure for changes due to ageing, where older dogs generally needed longer to learn the new concept; in the reversal training phase the dog needed to select the other (“reversed”, previously not rewarded) training stimulus [17,19]. Training protocols that include reversal learning often require days and hours of training. In the current study, time was a limiting factor. Being able to reach the training criterion and having the intervention within 30 min after that on the same day was crucial to avoid other potential influences/effects, posed by the fact that all dogs were pet dogs living with their owners in different households. Nevertheless, future studies that want to explore the stability over time of previously learned associations, by also including effects of age, should have a bigger sample size, and should also include reversal learning training tasks.

The findings of this follow-on study indicate that pleasant arousal post-learning has similar effects on enhancing memory in dogs as it does in humans [20]. Surprisingly, a recent study [5] failed to find a positive effect on memory consolidation when dogs were allowed to play with a Kong^®^ dog toy (Golden, CO, USA) and were retested on a newly learned task immediately afterwards. It was postulated that emotional arousal had a deteriorative effect. However, it can also be hypothesized that playful activities need to have a social element that includes playing with a human to improve the memorability of an event. Indeed, play is a regular feature of many dog–human interactions [21]. It has been shown that dogs prefer social play with humans over solitary play [22], and that dogs rarely play individually; rather they choose to play with humans [23]. Play is considered to be intrinsically rewarding [11], induces a positive affective state in both parts of the human–animal dyad, and, hence, can strengthen the human–dog relationship [24]. In addition, owners who petted, talked to, and played with their dogs, showed an increase in beta endorphins, prolactin, dopamine, and oxytocin [25], indicating the postulated positive affective state of play. In summary, it is suggested that self-directed object play (such as the Kong^®^ toy used by [5]) might not serve the same function in memory consolidation mechanisms when compared to human–dog play, as used in the present study. The hormone levels of the researchers and the dogs were not measured in the current study, but should be included in future studies to elucidate this topic further.

Another important aspect of this study is that resting after the initial task acquisition did not negatively interfere with memory consolidation. All dogs were able to finish the re-training successfully, both after 24 h and also after one year later. Resting for 30 min included periods of lying down and/or walking around to settle somewhere else in the room, but dogs were not allowed to fall asleep. This is an important aspect, as it has recently been shown that a period of sleep can positively affect memory consolidation in dogs [2,5]. The strongest effect of sleep was found only when lasting over 3 h or when having a period of overnight sleep, and re-testing was done after one week of the initial task acquisition [2,5]. Hence, an additional positive effect of overnight sleep on memory in both training groups cannot be excluded.

In the first study, the same researcher conducted both the training and the interventions, which might have led to the researcher and/or the testing location being positively associated with a reward (i.e., sausage treats in the testing room, playful interaction with the researcher). This would mean that if the dog–human play intervention was positively associated with either the researcher or the location itself, serving as positive reinforcement, the dogs’ performances in the playful activities group would have been inadvertently influenced. To account for these limitations of the initial study, the follow-on study was conducted in a different location (while still using the same set up), and with different researchers. The new location and the researchers were unknown to the dogs and their owners. Both the training location and the researchers had no significant effect on re-learning the task 1 year later.

Another major limitation of this study design is the lack of a control for physical exercise without an emotional component, as it has been shown that acute exercise impacts memory consolidation (see meta-analysis [26]). More specifically, exercise has been reported to have a positive impact on memory by increasing synaptic plasticity and long-term potentiation [27], both of which take place during the consolidation process. It has also been demonstrated that acute exercise post-learning improved memory recall of some training tasks in senior dogs [28]. For this reason, an exercise component of the dog–human play intervention contributing to the improved training performance cannot be excluded. However, a strong effect appears unlikely in dogs, given that on lead exercise for one hour failed to improve training performance when retested on a novel task on the same day in a different study [5].

Measuring average heart rate, which did not significantly differ between groups, controlled for general arousal level during memory recall. Hence, it is concluded that arousal levels per se during re-training after one year and at the time of recalling information [29] is unlikely to have affected memory formation and memory retrieval mechanisms in this follow-on study.

## 5. Conclusions

Playful activities with a human for 30 min post-learning affected long-term memory, not only after 24 h, but potentially also up to 1 year later. The results of this follow-on study warrant further investigations into how memory recollection and training performance in dogs can be optimized. Despite the low sample size, and its inherent limitation, so far dog–human play and structured periods of sleep seem to be the most promising training interventions. A better understanding of efficacious interventions (but also deleterious effects) would be of tremendous practical use, both in the professional and the private sector of dog training. Future studies should look into the potential synergistical effects of dog–human play (and other forms of positive affective states) when followed by a longer period of sleep. In addition, the effect of ageing on learning and memory should be addressed by recruiting more balanced age groups and by using more complex training tasks.

## Figures and Tables

**Table 1:** Key demographics of the retested individual dogs.

Number	Dog	Intervention	Trained Object	Sex	Age (Years)
1	Max	Rest	basket	m	8
2	Poppy	Play	basket	f	3
3	Hope	Rest	box	fs	8
4	Kess	Play	box	fs	4
5	Meg	Play	box	fs	7
6	Moya	Rest	box	f	3
7	Poppet	Rest	basket	fs	6
8	Penny	Play	basket	f	3
9	Bramble	Play	basket	fs	4
10	Bruno	Rest	box	mn	10
11	Monty	Rest	basket	mn	7

Basket: dogs being trained to go to a blue, dotted, woodchip filled basket; box: dogs being trained to go to a green, striped, cat litter sand filled box; f: female; fs: female spayed; m: male; mn: male neutered.

**Table 2:** Training performance data of retested individual dogs.

Dog ID	Age (Years)	Inter-Vention	Mean Heart Rate ± SD	Number of Trials Day 1	Number of Trials Day 2	Number of Trials 1 y	Rel. Diff. 1 y to Day 2 (%)	Rel. Diff. Day 2 to Day 1 (%)	Rel. Diff. 1 y to Day 1 (%)
1	8	Rest	117 ± 55	40	47	47	0	18	18
3	8	Rest	80 ± 35	70	52	131	152	−26	87
6	3	Rest	118 ± 30	69	73	24	−67	6	−65
7	6	Rest	126 ± 19	140	59	37	−37	−58	−73
10	10	Rest	101 ± 35	84	23	39	70	−73	−53
11	7	Rest	106 ± 56	92	47	49	4	−49	−47
2	3	Play	85 ± 11	76	23	36	57	−70	−52
4	4	Play	103 ± 24	65	22	23	5	−66	−65
5	7	Play	125 ± 38	104	36	47	31	−65	−55
8	3	Play	94 ± 19	37	22	20	−9	−40	−46
9	4	Play	97 ± 16	63	24	24	0	−62	−62

Rest: resting group; Play: playful activities group; SD: standard deviation; Rel. diff.: calculated relative difference of trials needed to meet training criterion in percent (%); y: year; -: relative reduction in trial number in percent (%).
